# Integration of an Electronic Screening, Brief Intervention, and Referral to Treatment Program Into an HIV Testing Program to Reduce Substance Use and HIV Risk Behavior Among Men Who Have Sex With Men: Protocol for Intervention Development and a Pilot Randomized Controlled Trial

**DOI:** 10.2196/56683

**Published:** 2024-03-14

**Authors:** Iván C Balán, Ruben O Marone, Victoria Barreda, Sylvie Naar, Yuxia Wang

**Affiliations:** 1 Center for Translational Behavioral Science Department of Behavioral Science and Social Medicine Florida State University College of Medicine Tallahassee, FL United States; 2 Nexo Asociacion Civil Buenos Aires Argentina

**Keywords:** HIV, substance use, community health, implementation science, eHealth

## Abstract

**Background:**

Men who have sex with men (MSM) are disproportionally affected by HIV and drug and alcohol use; however, few effective HIV prevention interventions for MSM who use substances exist. Screening, Brief Intervention, and Referral to Treatment is an early intervention for non–treatment-seeking individuals with problematic substance use and for timely referral to treatment for those with substance use disorders. Electronic screening and brief interventions (e-SBIs) reduce implementation challenges. An e-SBI tailored for MSM at the time of HIV testing might be particularly opportune to strengthen their motivation to reduce substance use and HIV risk behavior.

**Objective:**

This study aims to develop a tailored e-SBI program to reduce substance use and HIV risk behavior among MSM seeking HIV testing at Nexo Asociación Civil, our community partners in Argentina (primary); assess the feasibility and acceptability of integrating the e-SBI into the Nexo HIV testing program (primary); assess the feasibility and acceptability of implementing an adapted Men’s Health Project (MHP) at Nexo (secondary); and finally, explore preliminary findings on substance use and sexual risk reduction outcomes (exploratory).

**Methods:**

This mixed methods study has 2 stages. During stage 1 (development), we will use the User Centered Rapid App Design process consisting of focus groups (n=16), individual interviews (n=24), and a pilot deployment of the e-SBI (n=50) to iteratively develop the e-SBI. Quantitative and qualitative assessments at each step will inform the revision of the e-SBI. Furthermore, we will use the assessment, decision, administration, production, topic experts, integration, training, testing framework to adapt MHP. During stage 2 (pilot randomized controlled trial [RCT]), we will randomize 200 MSM coming to Nexo for HIV testing. They will complete a baseline assessment and then their assigned intervention (e-SBI vs screening only) and will be followed-up for 6 months. We will also conduct in-depth interviews with up to 45 participants: 15 participants from either study condition who entered or completed MHP or other substance abuse treatment and 15 from each arm who met the criteria for MHP but did not request it.

**Results:**

The study began recruitment in October 2022, and the stage-1 pilot study is near completion. Preliminary findings from stage 1 show high e-SBI acceptability. Data analysis of the stage-1 pilot is now beginning. The stage-2 pilot RCT will be launched in March 2024, with all data collection completed by May 2025.

**Conclusions:**

This study will allow us to assess the acceptability and feasibility of e-SBI implementation during HIV testing encounters. We will also build the necessary research infrastructure for a subsequent RCT to assess the efficacy of e-SBIs in reducing substance use and HIV sexual risk behavior among MSM in this setting.

**Trial Registration:**

ClinicalTrials.gov NCT05542914; https://tinyurl.com/yyjj64dm.

**International Registered Report Identifier (IRRID):**

DERR1-10.2196/56683

## Introduction

### Background

Men who have sex with men (MSM) are disproportionally affected by HIV and drug and alcohol use [1-11]. Given the association between substance use and HIV risk behavior among MSM [12-25], the dearth of effective HIV prevention interventions for MSM who use substances is striking. However, as in the general population, identifying and engaging MSM with problematic substance use (PSU) in treatment is a significant challenge [26-28] that results in most MSM with PSU never receiving treatment [29].

The Substance Abuse and Mental Health Services Administration recommends Screening, Brief Intervention, and Referral to Treatment (SBIRT) as an early intervention for nontreatment-seeking individuals with risky alcohol and drug use and for timely referral to more intensive treatment for those with substance use disorders [30]. However, implementing SBIRT is a challenge; brief intervention (BI) is often missed and not evidence based, which can diminish its effectiveness. For example, when SBIRT was implemented in sexually transmitted disease clinics in New York City, out of 66,989 positive screens, only 17,474 received BI [31]. In medical settings, factors such as limited staff time, commitment to implementation, training requirements, difficulty in learning evidence-based BIs, skepticism about the effectiveness of SBIRT, and poor fidelity to recommended BI guidelines are all obstacles to the successful implementation of SBIRT [32-39]. These factors limit the BI in a particular setting. BIs can range from providing normative feedback to the individual on their level of severity (at times just a written report), brief advice (10-15 min), or a single 30- to 45-minute session. However, studies have shown that interventions that go beyond normative feedback and provide an empathic [33] or motivational component [34], or consist of multisession interventions for high-risk users, have better outcomes but can be difficult to implement. As a result, sizeable percentages of patients in a setting go unscreened, those who meet the criteria for a BI may not receive it, the BI may be delivered inconsistently, and the BI may not be of sufficient intensity to reduce substance use among those with low or moderate risk use or to improve entry into substance abuse treatment among those with high-risk use or dependence [35].

Electronic screening and brief interventions (e-SBIs) reduce implementation challenges such as extensive training, demands on staff time, and inconsistency in the delivery of BI [32,36-42], while remaining as effective as in-person SBIs [43-46]. Because an e-SBI demands only that a staff member provide an individual with a tablet or computer space to complete both the screening and BI, more individuals can be screened and receive the BI. For example, in an implementation pilot study that used e-SBI in rural clinics, approximately 92% of patients were screened, with over 95% of screen-positive women receiving the tablet-based BI, which far exceeds the rates in trials of person-delivered SBIRT (SJ Ondersma, personal communication). Furthermore, e-SBIs are consistently rated highly for acceptability among users and may facilitate greater disclosure of PSU than in-person SBIs because of concerns of being judged by providers. As such, an e-SBI tailored for PSU and sexual risk behavior among MSM at the time of HIV testing, when a contemplative client is already concerned about his risk behavior and cares enough about his health to seek an HIV test, might be particularly opportune to strengthen his motivation to reduce substance use and HIV risk behavior.

The need for such interventions is particularly acute in Argentina and Latin America, where there is little implementation of evidence-based interventions for MSM with PSU. In *Proyecto LINKS*, our study of 500 MSM in Buenos Aires [47], 61% of the men reported drug use and over 40% reported polydrug use during the previous 2 months. Marijuana, tranquilizers, cocaine, and *pasta base* (cocaine sulfate) were particularly prevalent. Furthermore, over 20% of users of these substances used them daily [9]. Most participants in the study consumed alcohol during the past 2 months: 32% of them reported drinking at least weekly and drinking enough to “feel it a lot,” “get drunk,” or “feel like you might pass out.” Substance use was higher among participants who were younger, unemployed, had greater mood variability, and did not identify as gay [9]. HIV prevalence, at 17% (31% among gay-identified men), and incidence, at 4.53 per 100 person-years (5.60 per 100 person-years in gay-identified men), were also alarming [48]. These findings highlight the critical need for effective, evidence-based HIV prevention interventions for substances using MSM in Argentina.

The proposed study seeks to address this glaring gap by using a user-centered process to develop and pilot a tablet-based e-SBI program tailored to MSM awaiting their HIV test at Nexo Asociación Civil, our community partners in Buenos Aires, who conduct over 1500 HIV tests per year with MSM. The e-SBI will integrate substance use and sexual risk behavior screeners and individually tailored motivational interviewing (MI) as the BI. Furthermore, we will adapt and pilot the implementation of the Young Men’s Health Project (YMHP) [49], a 4-session MI-based intervention that effectively reduced substance use and condomless anal intercourse among MSM who use substances, as a brief treatment provided at Nexo for participants with moderate-risk substance use or to build motivation to enter specialized substance abuse treatment among those with high risk or dependence.

### Study Objectives

As a National Institutes of Health R34 (Clinical Trial Planning Grant) study, the main goal of the study is to develop and pilot all the study components and build the necessary research infrastructure for a subsequent randomized controlled trial (RCT) to assess the efficacy of the e-SBI to reduce substance use and sexual risk behavior. The specific aims of this R34 study are as follows:

1. Develop a tailored e-SBI program to reduce substance use and HIV risk behavior among MSM seeking HIV testing
2. Conduct a pilot RCT of e-SBI versus screening only (3:1 ratio) to assess the acceptability and feasibility of integrating e-SBI into the Nexo HIV testing program and the feasibility of a future large-scale efficacy trial of e-SBI, as measured by the following:
  Percent of MSM testing clients at Nexo who accept entry into the study (recruitment rate)
  Percent of participants who complete the e-SBI (retention rate)
  Retrospective acceptability ratings of the 2 RCT conditions (primary)
3. Assess the feasibility and acceptability of implementing the Men’s Health Project (MHP) at Nexo, as measured by the following:
  The percentage of MSM with moderate or high-risk substance use who enter MHP from either RCT condition (recruitment rate)
  The percentage of MHP participants who complete all 4 sessions
  Prospective acceptability of MHP among participants in either RCT condition
  Retrospective acceptability of MHP among those who received it
  Percentage of sessions conducted by each MHP counselor that meet the criteria for MI proficiency on the Motivational Interviewing Treatment Integrity (MITI.4) ratings

## Methods

### Study Design

This 3-year study began on August 1, 2022, and comprises 2 stages. The first stage, *Development*, will consist of developing the e-SBI, adapting the YMHP into MHP, training counselors to deliver MHP, and piloting the e-SBI with 50 MSM coming to Nexo for HIV testing. During the second stage, *the Pilot RCT*, we will randomize 200 MSM coming to Nexo for HIV testing at a 3:1 ratio (e-SBI to screening assessments only) to assess the feasibility and acceptability of the e-SBI among MSM coming to Nexo for HIV testing and establish and pilot the RCT process for a future trial. As a secondary aim, we will assess the uptake, acceptability, and feasibility of delivering MHP to participants with moderate-risk substance use and subsequent referrals to substance abuse treatment among participants with high-risk substance use or dependence. Finally, we will explore the preliminary findings on substance use and sexual risk reduction outcomes.

At the outset of the study, we will convene a Community Advisory Board (CAB) composed of 6 members, including representatives from a local community-based organization focused on policy, training, and social marketing regarding substance abuse; a representative from the Department of Sexual Health, AIDS, and STIs at the Ministry of Health of the City of Buenos Aires; and 4 MSM who report using drugs and alcohol recreationally (these criteria will not be known to others). The CAB will meet study investigators (in person or via videoconferencing) quarterly throughout the study. The CAB will be responsible for reviewing and providing feedback on the e-SBI, MHP, and assessment instruments that will be used in the study before they are used with the study participants.

This study was registered at ClinicalTrials.gov on September 16, 2022. The identifier number was NCT05542914.

### Ethical Considerations

#### Ethics Committee Approvals

The study was reviewed and approved by the Florida State University Institutional Review Board (IRB ID: STUDY00002530) and the Ethics Committee of Nexo Asociación Civil (protocol 26082022).

All participants were provided with a written informed consent form to read before participating in the study. The consent form included an overview of the study, study procedures, risks of participation, procedures implanted to minimize risks of participation, a statement of voluntary participation, and contact information for the principal investigators in Argentina and the United States, along with contact information for the respective institutional review boards. Consent was documented with the signature of the participants and research staff.

The results of the study may be published or presented, but no information that can identify individual participants will ever be provided or released in publications or presentations. To protect the privacy and confidentiality of participants, the study staff will keep any records containing identifying information in a locked cabinet accessible only to the research staff. The computer programs used in tablet-based interventions are compliant with US regulations on personal health information, as are the servers on which the data are stored. All data captured on the tablet, although it does not include identifying information, are encrypted in transit to maintain confidentiality. Any other study data will be stored on a secure computer that is protected by passwords. All records will be kept confidential. This is consistent with the guidelines of the US National Institutes of Health. After removing all identifying information, the remaining deidentified information collected during the study could be used for future research studies or distributed to another investigator for future research studies without additional consent.

All participant incentives will be paid in cash in Argentine pesos. In stage 1, focus group (FG) participants will receive US $25; individual interview participants will receive US $20; and e-SBI pilot participants will receive US $10 and an additional US $10 for those who complete a qualitative interview. In stage 2, RCT participants will receive US $15 for completing the baseline assessment and e-SBI screeners, US $15 for the month 3 follow-up assessment, and US $20 for the month 6 follow-up assessment. Participants who complete the 6-month in-depth interview (IDI) will receive an additional US $20.

### Components of the Intervention

#### MI Intervention

MI is the basis for both the e-SBI and MHP. MI is “a collaborative, goal-oriented method of communication aimed at strengthening an individual’s motivation for and movement toward a specific goal by eliciting and exploring the person’s own arguments for change.” [50] In an MI session, a clinician guides rather than pushes a client toward change, sidestepping resistance, and actively works with the patient’s strengths to build self-efficacy toward the desired outcome. MI uses standard tools of counseling and psychotherapy (ie, open-ended questions, affirmations, reflections, and summaries) strategically to elicit and reinforce change talk (patient statements that argue against the status quo, such as “I have been doing crazy things when I use drugs,” or for behavior change, such as “I want to have sex sober so I don’t want to worry the next day about whether I got HIV”) and to inhibit sustain talk (patient statements that argue against change, such as “Sex a little high is fun”). Process research on the mechanisms of action in MI shows that more change talk [51,52] and less sustained talk during a session are related to better outcomes [51,53]. In addition, counselor behaviors such as advising, confronting, directing, and warning clients are associated with greater sustained talk [54,55], whereas affirming a client’s strengths or effort, emphasizing client control, and supporting clients are associated with increased change talk [55,56]. Thus, to facilitate behavior change, the goal of an MI counselor is to interact with the client in a way that fosters change talk and inhibits sustain talk [51]. These key aspects of MI will inform the programming of the narrator in the e-SBI (described below), the revision of MHP, and the training of MHP counselors. The e-SBI will be created using the Computerized Intervention Authoring System (CIAS) V.3, which is an authoring tool that allows the creation and editing of electronic screening and intervention packages without the need for a programmer. Interventions built using CIAS feature a synthetic text-to-speech engine that reads all questions and speaks aloud to the participant (via headphones); synchronous interactivity; natural language reflections; branching logic; a clean user interface; and the ability to easily incorporate images, text graphs, figures, or videos. CIAS is consistent with human-computer interaction literature, suggesting that interactive, lifelike software can engage users and promote behavior change [57-61]. Thus, CIAS uses an optional interactive cartoon character capable of over 50 specific animated actions (smile, wave, read a message, express concern, etc) that talk for the entire program. The character acts as a narrator and guide throughout the process and provides occasional comic relief. Everything the character does is specifically programmed during the development phase, including interacting with participants in an MI-consistent manner and reflecting participants’ change talk derived from their responses to the assessments. For example, using the participants’ responses, the narrator might be programmed to say “You don’t drink very often, but when you do, you drink a lot (from Alcohol Use Disorders Identification Test [AUDIT] responses) and you are noticing that it is impacting your physical health and your relationship with others (from responses to the Short Inventory of Problems).” CIAS has been used with thousands of participants (primarily those with low socioeconomic status) and has consistently received high satisfaction ratings [62-66].

#### YMHP Intervention

YMHP is a 4-session MI-based intervention that was the first intervention to demonstrate reductions in substance use and sexual risk behavior among non–treatment-seeking MSM who use substances [49]. Compared with the time- and content-matched control group, YMHP recipients were 18% less likely to use drugs and 24% less likely to engage in unprotected anal intercourse. On days when drug use and sex co-occurred, drug use reductions led to a decrease in unprotected anal intercourse. Secondary analyses showed that drug use among partnered participants was stable but declined significantly at all follow-up time points among single men [67]. We will adapt and implement YMHP as an in-house brief treatment for participants with PSU, either as a stand-alone treatment (for those with moderate-risk use) or as an intervention to link those with high-risk use or dependence on substance abuse treatment.

### Stage 1: Development

#### Overview

The development of the e-SBI app will be guided by the User Centered Rapid Application Development (UCRAD) process [68-72]. UCRAD merges the streamlined and iterative Rapid Application Development approach with a user-centered design approach, which engages intended users throughout the app development process. This integrated approach aims to develop successful apps with good functionality, simple features, and usable interfaces [69]. As such, the intended users are given access to prototypes of the app, allowing them to provide feedback before the next iteration of the app. The process is relatively brief, approximately 6 to 9 months. UCRAD uses a 3-phase process as follows:

1. Predesign and interface prototyping: Data are gathered from key stakeholders to understand the requirements of the app in terms of content and interface. While continuing to assess user needs, developers offer potential solutions for the design. At the end of this stage, an initial prototype is developed based on the initial requirements.
2. System architecture and coding: The prototype is shared with the intended end users to obtain feedback. The developers update the prototype according to the feedback provided and begin system coding to develop a more complete version of the app. This version is returned to users for another round of feedback and development. The result of this stage is an advanced but often incomplete product with basic system architecture and system specifications.
3. Deployment: The app is implemented in the field to obtain further feedback from potential end users, and further development of the application is conducted to reach a final product.

#### Predesign and Interface Prototyping

To accomplish this phase, we will obtain feedback from the CAB regarding the study screening instruments and preliminary plans for the e-SBI content. After incorporating their feedback, we will conduct 2 FGs with MSM (n=16) recruited from Nexo testing clients who were 18+ years of age and self-identified as male. They will complete a substance use screener consisting of an illicit drug use question on the National Institute on Drug Abuse–modified Alcohol, Smoking and Substance Involvement Screening Test (ASSIST) brief screen and the AUDIT-C (a 3-item alcohol use screen). Those who indicate using illicit drugs at least monthly or receive a score of ≥4 on the AUDIT-C will be eligible to participate. After obtaining informed consent, they will complete paper and pencil versions of the screening instruments planned for inclusion in the e-SBI, including:

- The *National Institute on Drug Abuse–modified ASSIST* [73,74] substance use assessment, which has high consistency in detecting moderate and high substance use, and high concordance (90%-98%) and correlation (intraclass correlation coefficient 0.90-0.97) for tobacco, alcohol, and drug risk scores [75]. A self-administered computer ASSIST was found to have a 0.93 interclass correlation with the interviewer-administered version [76].
- *AUDIT* [77,78] is a widely used tool developed by the World Health Organization to identify individuals whose alcohol consumption has become hazardous or harmful to their health. AUDIT is a 10-item screening questionnaire with 3 questions on the amount and frequency of drinking, 3 questions on alcohol dependence, and 4 on problems caused by alcohol.
- *Short Inventory of Problems-Alcohol and Drug Use (SIP-AD) for MSM* [79] is a 10-item version of the SIP-AD [80] (ɑ=.95) derived through Item Response Theory, which was found to be highly valid and reliable among non–treatment-seeking MSM. The SIP-AD assesses the frequency (0=never; 3=daily or almost daily) of commonly experienced consequences of alcohol or drug use across the physical, intrapersonal, interpersonal, social responsibility, and impulse dimensions.
- *Sexual Practices Assessment Schedule* [81] asks about the number of partners and occasions of oral, anal, and vaginal sex with male, female, and transgender sexual partners in the past 2 months; use of condoms during sexual intercourse; and assumed HIV status of the partner.
- *Readiness to Change Rulers* for alcohol use, risky sexual behavior, and drug use: although they vary in their specific wording, single-item readiness to change rulers that ask respondents to place themselves on a scale of change from 1-7 or 1-10 have shown concurrent and predictive validity for alcohol use [82-85], drug use [82,86-88], and sexual risk behavior [84,89]. These scales are also often used in MI interventions to strategically evoke change talk and increase motivation to change problem behaviors.

The 90-minute audio-recorded FGs will (1) explore participants’ reactions to the screeners and their impact on risk perception and motivation to reduce PSU and risk behavior and (2) evoke content for the BI component to develop options for reasons to change and potential approaches to reduce PSU and sexual risk behavior from which e-SBI users can choose. Within 48 hours of completing the FG, we will initiate a rapid analysis of these data [90,91] using a qualitative matrix [92,93] onto which participants’ responses and recommendations will be entered to quickly organize and summarize FG findings. These findings will be used to build a prototype of the e-SBI.

#### System Architecture and Coding

**Figure 1:**
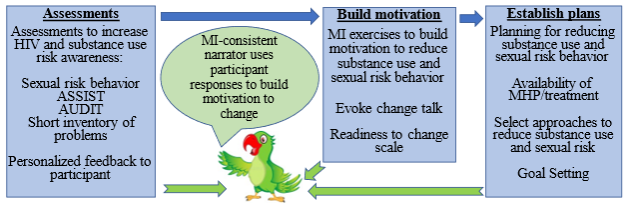
Conceptualization of the electronic screening and brief intervention (e-SBI) intervention to reduce substance use and HIV sexual risk behavior among men who have sex with men in Argentina; ASSIST: Alcohol, Smoking and Substance Involvement Screening Test; AUDIT: Alcohol Use Disorders Identification Test; MI: motivational interviewing; MHP: Men’s Health Project.

This iterative app development process will be conducted with waves of 6 MSM recruited from Nexo testing clients, with the same inclusion criteria as in the FGs. They will be recruited during 2-week intervals to maintain a steady pace of feedback and revisions. The participants completed the intervention on the tablet. They then complete a 7-item self-report instrument used by CIAS developers [64] that assesses how much participants (1) liked the e-SBI and fount it to be (2) interesting, (3) easy to use, (4) made up of understandable questions, (5) respectful, (6) annoying, and (7) humorous; each item is rated from 1=not at all to 5=very much. Afterward, participants will be debriefed during an audio-recorded interview to explore (1) their responses to the acceptability assessment; (2) overall reactions to e-SBI; (3) emotional reactions as e-SBI progressed, including heightened risk awareness, concern, or motivation to reduce substance use and sexual risk behavior; and (4) recommendations for improving the ability of the e-SBI to engage MSM with high-risk drug and alcohol use in treatment. The interview data will undergo the same matrix-based, rapid analysis process used for the FG to quickly inform the next revision of the e-SBI. This process will be repeated with waves of 6 participants until mean scores on each acceptability question are ≥4.0 (≤2.0 on annoying), which we expect within 3 to 4 cycles. Figure 1 depicts our current conceptualization of the e-SBI, with specific content informed by the stage-1 findings. The development of the e-SBI will be guided by Choice Theory [94], which has been used extensively to inform goal choice interventions [95-100], including MI interventions to reduce substance use among MSM [97,98]. Rather than preset outcome expectations (ie, abstinence), goal choice interventions highlight personal choice and goal setting to engage individuals in reducing drug and alcohol use. Goal choice interventions are equally effective as abstinence-based interventions, allowing individuals to set their own treatment goals, which appears to increase success rates [99,100].

#### Deployment

The initial version of the e-SBI will be deployed with 50 MSM coming to Nexo for HIV testing to be used while awaiting their HIV test. On the basis of the findings from LINKS and Nexo screening data, we estimated that at least 50% will report using drugs and 80% will report using alcohol, and that 20% to 25% will report PSU. As such, a sample of 50 will allow us to explore reactions to the e-SBI from MSM with no, low-risk, and moderate to high-risk substance use. After obtaining informed consent, these individuals will complete the e-SBI and a brief quantitative acceptability assessment and then proceed to undergo their HIV test (to minimize participant distress). Subsequently, up to 25 of these participants will be randomly selected (5 with low-risk substance use and 20 with moderate or high-risk substance use) to undergo an audio-recorded IDI that will be guided by the theoretical framework of acceptability (TFA; see components in Textbox 1) [101], which has been used to guide qualitative and mixed methods research on the acceptability of health care interventions [102-105]. Data from these interviews will also undergo a rapid qualitative analysis as previously described for the FGs.

**Components of the theoretical framework of acceptability.**

- *Affective Attitude:* how the individual feels about the intervention
- *Burden:* perceived amount of effort that is required to participate in the intervention
- *Ethicality:* extent to which the intervention has a good fit with an individual’s value system
- *Intervention Coherence:* extent to which the participant understands the intervention and how it works
- *Opportunity Costs:* extent to which benefits, profits, or values are given up to engage in the intervention
- *Perceived Effectiveness:* extent to which the intervention is perceived as likely to achieve its purpose
- *Self-efficacy:* the participant’s confidence that they can perform the behaviors required to participate in the intervention

#### Adaptation of YMHP to MHP

In consultation with our CAB, the research team will adapt YMPH while guided by the modified ADAPT-ITT (assessment, decision, administration, production, topic experts, integration, training, testing) framework (Textbox 2) [106]. The adaptation will focus on three main areas: (1) tailoring YMHP to Argentine MSM (including translation and cultural relevance for MSM aged over 29 y); (2) adapting YMPH from a stand-alone intervention to an intervention within an SBIRT program by minimizing duplication with e-SBI content and incorporating content to improve linkage to specialized substance abuse treatment for participants with high-risk substance use or dependence; and (3) adding content to address its poorer efficacy among partnered MSM.

**Overview of the ADAPT-ITT (assessment, decision, administration, production, topic experts, integration, training, testing) framework.**

- Assessment phase: completed as part of our earlier studies and discussions with stakeholders
- Decision phase: completed as part of our earlier studies and discussions with stakeholders
- Administration phase: perform theater test to obtain feedback
- Production phase: produce revised intervention, maintain fidelity to core elements and underlying theory. Develop quality assurance and process measures
- Topic experts phase: involve topic experts in adapting the intervention
- Integration phase: integrate content from topical experts; integrate new assessments based on revised content
- Training phase: train staff to deliver the intervention
- Testing phase: test revised intervention in pilot study

#### Administration

To obtain feedback from members of the target population and key stakeholders, we video recorded a mock YMHP session (conducted in Spanish) that will be shown to the CAB, Nexo counselors (most of whom identify as gay men), and study coinvestigators. As they watch the videos, audience members will be asked to complete a feedback form, divided by portions in the session, so that they can provide feedback on issues they felt needed to adapt from the original. Next, we will discuss recommendations for addressing the issues raised.

#### Production

Feedback obtained during the administration phase will inform draft 1 of MHP. The revised intervention will be translated into Spanish and manualized to standardize counseling sessions and provide support and guidance for counselors.

#### Topic Experts and Integration

Draft 1 of MHP will be reviewed by substance abuse experts on the CAB, Nexo counselors, and coinvestigators to identify any other components of the intervention that need revision. Feedback from this review will be integrated into the intervention to create draft 2, which will be pilot-tested during the stage-2 RCT.

#### Training of MHP Counselors

The training of MHP counselors will be consistent with research on learning MI, which recommends a 2-day workshop with follow-up feedback and coaching [107,108]. As such, we will conduct a 4-day training with MHP providers; the first 2 days will focus on general MI principals and skills and the last 2 days will focus on specifics of MHP. Counselors will then conduct at least 2 mock cycles of MHP, which will be audio recorded and uploaded to a secure internet site for the MI trainer to rate using the MITI.4 scale [109], which captures the counselor’s level of fidelity to MI and establishes specific criteria for *Proficiency* in MI. These ratings will be used to assess MI skills that require further development, which will guide videoconference coaching sessions.

#### Testing

Although the primary aim of this study was to focus on the e-SBI component, as a secondary aim, we will assess the potential uptake and acceptability of MHP during the stage-2 pilot RCT.

### Stage 2: Pilot RCT

#### Overview

**Figure 2:**
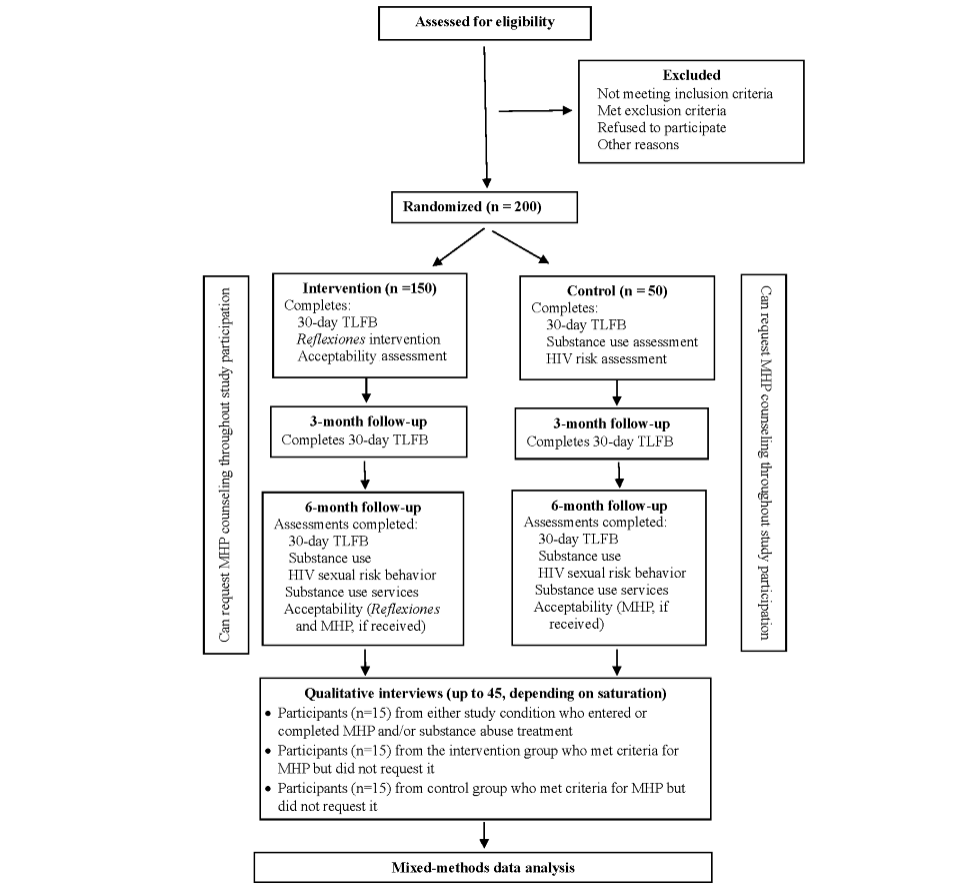
CONSORT (Consolidated Standards of Reporting Trials) diagram for the proposed phase-2 pilot randomized controlled trial of electronic screening and brief intervention (Reflexiones). MHP: Men’s Health Project; TLFB: Timeline Followback.

With the e-SBI finalized, MHP adapted, and MHP counselors trained, the study will proceed to the stage-2 RCT comparing e-SBI to screening assessment only (see the CONSORT [Consolidated Standards of Reporting Trials] diagram for an overview; Figure 2). We will assess the feasibility and acceptability of e-SBIs and conduct a preliminary exploration of substance use and sexual risk reduction outcomes. On the basis of the findings from LINKS and Nexo data, we estimated that 50% of participants will report using drugs and 80% will report using alcohol, and that 20% to 25% will report moderate- or high-risk use based on the ASSIST and AUDIT criteria, respectively. As a secondary aim, we will assess the uptake, acceptability, and feasibility of delivering MHP at Nexo and the subsequent linkage to substance abuse treatment among those with high-risk substance use or dependence. The RCT process is described below.

#### Recruitment

To ensure that the flow of participants is manageable during the study in the context of an HIV testing program, we will recruit up to 4 participants per day, 3 days per week, adjusting as necessary based on recruitment patterns to ensure the recruitment of all 200 participants. MSM who come to Nexo for HIV testing and are 18 years of age or older will be offered participation in the study. These clients will be given a flyer that, consistent with goal-choice interventions, will create a low threshold for study participation by stressing personal choice, goal selection, and an intervention tailored specifically for MSM to determine how to best reduce behavior that puts them at risk of HIV infection. Clients who wish to participate will complete informed consent, be randomized immediately, and be given a tablet logged on to their respective study arm (and disposable headphones) to complete their study procedures. Clients who declined participation will undergo the standard HIV testing process at Nexo. The research staff will record the number of clients who refused to participate.

#### Baseline Assessment

Participants in both conditions will complete a demographics questionnaire and then a self-administered, tablet-based 30-day *Timeline Followback (TLFB)* [110] to track days of drug use; number of alcoholic drinks; and HIV risk behavior, defined as anal sex with (1) an HIV-positive partner who they were not sure had an undetectable viral load or (2) a partner who had not tested HIV negative within the past 3 months. The TLFB has demonstrated good test-retest reliability, convergent validity, and agreement with collateral reports for daily drinking [111] and sexual behavior [112]. The TLFB will be used to assess primary substance use and sexual risk behavioral outcomes (which are exploratory outcomes in this study). After completing the e-SBI, participants in the intervention condition also completed a brief quantitative assessment of acceptability (8 items) based on the TFA [113].

#### RCT Study Conditions

Participants will be randomly assigned into 1 of 2 study arms.

##### Intervention Group Participants

After completing the baseline assessment, the tablet will immediately lead these participants to the e-SBI, as finalized in stage 1. The CIAS branching logic allows the e-SBI to tailor the BI based on factors such as participants’ substance use risk level (based on ASSIST and AUDIT scores), risk perception, and readiness to change. Thus, BI may range from affirming and sustaining motivation among those with low-risk substance use to actively motivating and encouraging (in an MI-consistent manner) those with moderate or high-risk use to speak with a research assistant to obtain an appointment for MHP. The e-SBI will also target the participant’s sexual risk behavior and help them develop a risk-reduction plan, regardless of their substance use. Then, they proceed to the usual HIV testing process at Nexo.

##### Control Group Participants

After completing the baseline assessment, the tablet will lead these participants to the same screening instruments as in the e-SBI, but with no MI-based BI. Due to ethical considerations, control group participants with moderate- or high-risk substance use based on the ASSIST or AUDIT scores will receive brief feedback on the tablet stating their level of risk, availability of MHP at Nexo, and instructions to see the receptionist for an appointment, if desired. Then, they proceed to the usual HIV testing process at Nexo.

#### Referral for MHP

All individuals with moderate- or high-risk substance use will be recommended for MHP (either *actively motivated* to enter MHP through the MI component of the e-SBI for those in the intervention condition or recommended to enter treatment and made aware of the availability of treatment at Nexo for those in the control condition). MHP sessions will be conducted in-person at Nexo (later sessions can occur remotely, based on the participant’s preference) and will follow the intervention manual as finalized at the end of stage 1, with sessions expected to be 60 minutes in duration. As in the YMHP efficacy study, the 4 MHP sessions can be conducted over a period of up to 2 months. To maximize retention, participants will be offered reminder calls or text before each scheduled visit. For participants with moderate-risk substance use, MHP focuses on reducing substance use and sexual risk behavior. For participants with high-risk use or dependence, MHP sessions focus on building motivation to enter substance abuse treatment. After accepting the referral, the participant and Nexo staff contact the treatment provider to obtain an appointment at the clinic of the participant’s choice (ie, closer to home). Nexo staff will offer to accompany the participant to the appointment to provide a *warm handoff* to facilitate linkage to care. To ensure intervention fidelity, all MHP sessions will be audio recorded, uploaded to a secure website, and rated using the MITI.4. MITI.4 ratings will be conducted throughout the study and used to inform weekly videoconferencing coaching sessions for MHP counselors to improve and sustain MI skills.

#### Follow-Up Assessments

All participants, including those who enter MHP or substance abuse treatment, will repeat the self-administered 30-day TLFB at 3 and 6 months after enrollment to assess drug use, alcohol use, and sexual risk behavior. At 6 months, they will also complete the screener instruments in the e-SBI and TFA-based quantitative retrospective acceptability assessments of e-SBI and MHP (if they enter MHP), all of which will be self-administered. We will also conduct IDIs with up to 45 participants (or less if saturation is reached): 15 participants from either study condition who entered or completed MHP and substance abuse treatment and 15 from each arm who met the criteria for MHP but did not request it. Also guided by the TFA, we expect these IDIs to be similar to those conducted in the stage-1 e-SBI pilot; however, revisions will be made to the IDI guide based on how well the questions elicited the desired material from the stage-1 participants. The use of one framework to guide all of these IDIs and the qualitative data analysis will allow us to more rigorously compare participants with PSU who entered MHP and substance abuse treatment with those who did not to identify factors that might impact their decision to enter MHP. IDIs will be conducted at Nexo, audio recorded, and transcribed.

### Data Analysis

#### Qualitative Data Analysis for Stage-2 IDIs

For the stage-2 pilot RCT, transcripts from the audio recordings will be reviewed for accuracy and uploaded onto Dedoose for management and analysis. Codebook development will begin when 10 transcripts are available. First-level codes will be guided by TFA components, whereas second- and third-level codes emerge from themes identified in the narratives. An initial set of codes will be generated independently by 2 research staff members, compared, and then synthesized to compile shared coding categories and subcategories, all with definitions, inclusion and exclusion criteria, and examples. As transcripts become available, coding will continue, refining codebook definitions that will be used to process the remainder of the manuscript. The coders discussed discrepancies until they achieved 80% intercoder convergence. As per Patton [114], we will identify indigenous (ie, participant-generated) and analyst-constructed typologies. Once data are coded, analysis of coding reports will include categorization, abstraction, comparison (especially between groups in stage-2 RCT), integration, iteration, and refutation of themes.

#### Integration of Quantitative and Qualitative Data

We seek to exploit the richness of our mixed methods approach, with quantitative and qualitative findings enriching each other. Quantitative assessments allowed us to systematically explore variables of interest across all participants. Qualitative data will add nuances and new insights to quantitative findings. During data analysis, the interpretation of quantitative results will be enriched by the summary of codes for specific components of the e-SBI. In Dedoose, participants will be categorized based on their responses in the quantitative assessment (eg, severity of drug or alcohol use; ratings on readiness to change rulers) and use these categories to compare themes and concepts that emerge from the qualitative data.

#### Quantitative Data Analysis

##### Primary Aims

The primary aim of this study is to assess the feasibility and acceptability of integrating e-SBI into the HIV testing process at Nexo by estimating and comparing (1) the percentage of MSM testing clients at Nexo who accept entry into the study among those who are asked (recruitment rate); (2) the percentage of participants who complete e-SBI (retention rate); and (3) the mean score of 7-item e-SBI acceptability ratings between MSM participating in the e-SBI and those in the screening-only group. The secondary aim is to assess the feasibility and acceptability of implementing MHP at Nexo by estimating and examining the group difference with respect to (1) the percentage of MSM with moderate or high-risk substance use who enter or complete MHP; (2) percentage of high-risk enrollees in MHP who proceeded to substance abuse treatment; (3) mean acceptability of MHP and substance abuse treatment among those who received it (based on quantitative assessment to be developed); and (4) percentage of sessions conducted by each MHP counselor that meet the criteria for MI proficiency MITI.4 ratings. The estimation is accomplished by generating a point estimate with its corresponding 95% CI. We will use a 2-tailed *t* test for continuous outcomes and Fisher exact test for binary outcomes for the above comparisons. Adjustment for covariates, if necessary, with control baseline values, will be accomplished using multiple linear (continuous outcomes) or logistic (dichotomous outcomes) regression.

##### Exploratory Aims

Although this study did not recruit a sufficiently large sample to provide adequate power for assessing the efficacy of e-SBI, we will use data from the 30-day TLFB to explore changes in days of drug use, days of alcohol use, days of heavy alcohol use (5 or more drinks), and occasions of anal intercourse with a risky partner. To examine the efficacy of the above outcomes, a generalized linear model with a log link function was constructed, and generalized estimating equation methods were used to estimate model parameters and their SEs to account for correlation introduced by accessing the participants at multiple time points. Accompanying each of these tests will be point and interval estimates for the parameters of interest (ie, the ratio of the 2 group rate ratios). Although the hypotheses consider several simultaneous measures of effect, for the routine reporting of these results, there will be no adjustment for multiple comparisons.

##### Power Considerations

The primary goal of this pilot study was to collect preliminary data on the feasibility, acceptability, and target outcomes of the proposed intervention. We will use these pilot data to rule out unusually large or small true effects using standard 95% CI procedures. We confirm that the extrinsic effect sizes are contained within our CIs from the pilot. We examine the distribution of each variable and calculate the summary statistics by intervention condition. We will estimate key intervention parameters with sample means and proportions together with 2-sided 95% CIs and test the primary null hypotheses at the traditional 2-sided level α=.05 (simulation of the subsequent RCT). To plan the RCT, we will also consider 1-sided 90% confidence limits for mission-critical design parameters such as SDs and reference group end-point rates and proportions in the conservative direction. This is because we intend to plan sample size for the RCT so that power will be *excellent* (at least 80% power) for the clinically relevant effect size to be specified. This strategy will make proper allowance for the limited sample size of the pilot study with its consequent uncertainties and still yields appropriate sample sizes for the RCT. *Mission-critical* parameters include proportions for dichotomous variables (eg, recruitment rate and retention rate) and means and SDs for continuous outcomes (eg, days of drug use, days of alcohol use, acceptability of MHP and Substance abuse treatment). Large sample sizes are not required to locate these parameters approximately, but adequately, to plan the subsequent trial, whereas testing the study hypothesis in the pilot will generally not have sufficient statistical power. The estimation of mission-critical design parameters with point and CI estimates will be considered highly important; continuous outcome measures make it feasible to detect promising effect effects even in small samples. Means and SDs were estimated for continuous measures. For approximately normally distributed variables, an upper 1-sided 90% confidence limit for the SD, *sU*, was constructed from *sU* = *s* × *c2n,.10* × 1/2, where *s* is the sample SD and *c2n,.10* is the upper 10th percentile of the chi-square distribution with *n* degrees of freedom. For approximately log-normally distributed variables, logarithmic transformation was applied to achieve approximate symmetry and normality. For dichotomous variables (such as the primary and secondary outcomes or the PDR performance criteria), exact binomial methods will be used for the CIs for proportions. As an example, the width of the 95% CI around a point estimate of 50% (eg, MSM with moderate- or high-risk substance use who complete e-SBI and then enter MHP) would be 43% to 57% with 200 participants.

## Results

The study began with recruitment for stage 1 in October 2022. Currently, we are nearing the completion of stage 1. As part of the stage-1 development process, we conducted 2 FGs (n=16) and 2 waves of individual interviews (n=12). Consistent with the protocol, we ended the individual interviews after 2 of the 4 possible waves because acceptability scores were consistently high (mean score was >4 out of 5 for all participants). At present, we have almost completed the stage-1 pilot. At the conclusion of stage 1, we will conduct a rapid qualitative data analysis [90,91] to assess any additional changes that need to be made to the e-SBI before the stage-2 RCT, which we expect to launch in March 2024. At that time, we will also begin a deeper analysis of the stage-1 data from FGs, individual interviews, and the stage-1 pilot and include that in a future mixed methods manuscript to describe the intervention and its acceptability among the intended audience. We expect all data collection to be completed by February 2025 and the study results to be available by June 2025.

## Discussion

### Overview

This study addresses the critical need for interventions that reduce both PSU and HIV risk behavior among MSM in Latin America by adapting 2 evidence-based interventions, e-SBI and YMHP, for MSM in Argentina seeking HIV testing. Given that many MSM seek HIV testing in lesbian, gay, bisexual, and transgender (LGBT) community agencies, these interventions have the potential to reach large numbers of MSM, help them reflect on their use of drugs and alcohol, and help link those with PSU to evidence-based intervention (MHP). The provision of substance abuse treatment within an LGBT community agency can help facilitate entry and retention in treatment, as sexual minority men will not be concerned about the discrimination and microaggressions they might face at agencies serving the public. If proven efficacious in a future RCT, the implementation of these interventions in such agencies will help address a key contributor to HIV risk behavior and infection as well as reduce drug and alcohol use among MSM, who are disproportionately affected by HIV and substance use.

### Strengths

A key strength of this study is the iterative development of the intervention based on end user feedback to ensure its high acceptability among intended users. Feedback from end users is essential to create an intervention that achieves its aim of heightening risk awareness of PSU to motivate reductions in use in a respectful, nonjudgmental manner without creating resentment or defensiveness.

An additional strength is that the study and the intervention were developed in close collaboration with a community HIV testing center, where the intervention was designed to be delivered. Development within a community HIV testing program provides an early assessment of the implementation challenges that need to be overcome to maximize access to the intervention and minimize the burden on organizational processes and resources.

Finally, the e-SBI was developed using CIAS. As this system was designed for use by nonprogrammers, the CIAS minimizes costs for developing, revising, and maintaining electronic interventions. In addition, it was designed for long-term sustainability. For example, contrary to a custom-designed platform for a specific task, CIAS is a broad platform for use by many people; supporting only this single platform will automatically support the availability of everyone is work. Its ongoing development is still funded by the US National Institutes of Health for 2 years, and additional funding will be obtained to continue support. In addition, a yearly fee for funded projects provides income to support CIAS in the long term, especially given the high interest in its use. Finally, the source code will soon be made publicly available at no cost; therefore, it can be installed on a local server. This sustainability plan addresses a key challenge to other e-interventions that support falters once study funding has concluded, impeding the use of interventions in community settings.

### Limitations

This study and the intervention were designed in close collaboration with our community partners in Argentina. As such, there may be specific characteristics of our community partners and their client populations that may limit the generalizability of the findings to other community HIV testing programs. As a voluntary research study, HIV-testing clients with PSU may decide not to participate in the study, limiting the potential utility of this approach to identifying and engaging MSM with PSU to reduce their substance use. Finally, acceptability data (both quantitative and qualitative) are subject to social desirability. To minimize this risk, we will highlight for participants the importance of openly sharing their experiences and opinions, whether positive or negative.

### Conclusions

Completion of this Clinical Trial Planning Grant (NIH R34) will allow us to develop and pilot all the study components and build the necessary research infrastructure for a subsequent RCT to assess the efficacy of e-SBI in reducing substance use and HIV sexual risk behavior among MSM coming to a community agency for HIV testing.

## Appendix

Multimedia Appendix 1 Peer review reports.

## Abbreviations

ADAPT-ITT: assessment, decision, administration, production, topic experts, integration, training, testing
ASSIST: Alcohol, Smoking and Substance Involvement Screening Test
AUDIT: Alcohol Use Disorders Identification Test
BI: brief intervention
CAB: community advisory board
CIAS: Computerized Intervention Authoring System
CONSORT: Consolidated Standards of Reporting Trials
e-SBI: electronic screening and brief intervention
FG: focus group
IDI: in-depth interview
LGBT: lesbian, gay, bisexual, and transgender
MHP: Men’s Health Project
MI: motivational interviewing
MITI.4: Motivational Interviewing Treatment Integrity
MSM: men who have sex with men
PSU: problematic substance use
RCT: randomized controlled trial
SBIRT: Screening, Brief Intervention, and Referral to Treatment
SIP-AD: Short Inventory of Problems-Alcohol and Drug Use
TFA: theoretical framework of acceptability
TLFB: Timeline Followback
UCRAD: User Centered Rapid Application Development
YMHP: Young Men’s Health Project

## Data Availability

Once the study is completed, the data sets generated and analyzed during the study will be available from the corresponding author upon reasonable request. We will compile structured deidentified data sets and make them available for additional and secondary data analyses. For data sharing, the research team will follow the “standards for privacy of individually identifiable health information.” The principal investigator and research team will develop a process to review and approve data release based on concept sheets submitted by interested investigators and whether the data request is for new lines of analysis. Formal data-sharing agreements will be developed to guide and encourage further data mining of the proposed datasets for various purposes.
