# Supplementary material for: Integration of an Electronic Screening, Brief Intervention, and Referral to Treatment Program Into an HIV Testing Program to Reduce Substance Use and HIV Risk Behavior Among Men Who Have Sex With Men: Protocol for Intervention Development and a Pilot Randomized Controlled Trial
Source: JMIR Res Protoc. 2024 Mar 14;13:e56683. doi: 10.2196/56683 (PMC10979339; doi:10.2196/56683)
Supplement: Multimedia Appendix 1 [file resprot_v13i1e56683_app1.pdf]

SUMMARY STATEMENT
(Privileged Communication)

PROGRAM CONTACT:
MARCY Fitz-Randolph
301-443-9800
marcy.fitz-randolph@nih.gov

Release Date: **07/30/2021**
Revised Date:

Application Number: **1 R34 DA055503-01**

Principal Investigator

BALAN, IVAN C

Applicant Organization: FLORIDA STATE UNIVERSITY

Review Group: **HIBI**
HIV/AIDS Intra- and Inter-personal Determinants and Behavioral Interventions Study Section
AIDS - EXP. REV.

Meeting Date: **07/12/2021**
Council: **OCT 2021**
Requested Start: **12/01/2021**
RFA/PA: **PA18-780**
PCC: **CM/MEF**

Project Title: **Integration of Electronic SBI(RT) into an HIV Testing Program to Reduce Substance Use and HIV risk Behavior among MSM in Argentina**
SRG Action: **Impact Score:30**
Next Steps: **Visit https://grants.nih.gov/grants/next_steps.htm**
Human Subjects: 30-Human subjects involved - Certified, no SRG concerns
Animal Subjects: 10-No live vertebrate animals involved for competing appl.
Gender: 3A-Only men, scientifically acceptable
Minority: 5A-Only foreign subjects, scientifically acceptable
Age: 3A-No children included, scientifically acceptable

Project Year	Direct Costs Requested	Estimated Total Cost
1	125,000	176,604
2	175,000	247,245
3	150,000	211,924
TOTAL	450,000	635,773

ADMINISTRATIVE BUDGET NOTE: The budget shown is the requested budget and has not been adjusted to reflect any recommendations made by reviewers. If an award is planned, the costs will be calculated by Institute grants management staff based on the recommendations outlined below in the COMMITTEE BUDGET RECOMMENDATIONS section.

1R34DA055503-01 Balan, Ivan

RESUME AND SUMMARY OF DISCUSSION: This application proposes to develop a tablet-based screening (S) and brief intervention (BI) for MSM in Buenos Aires awaiting a HIV test at Nexo Asociacion Civil, a community organization. This electronic SBI will integrate sexual risk screening tests and substance use screening tests with an individually tailored Motivational Interviewing (MI) intervention that will seek to reduce substance use and condomless anal intercourse among MSM with moderate or high-risk substance use. After the intervention is developed and piloted with 50 MSM, 200 MSM awaiting HIV testing at Nexo Asociacion Civil will be randomly assigned to receive the intervention or to receive only screening assessments. In addition to evaluating the feasibility and acceptability of the intervention on study participants at low or moderate risk for substance use, the impact of the intervention on participants at high risk for substance use will be evaluated in terms of referrals generated for substance use treatment. There was a concern about the sustainability of the intervention in Argentina, but the committee also noted the rigor of the proposed study and the strength and expertise of the investigative team. The other main concern discussed by the committee was the feasibility of the proposed study given that the tight timeline will require the adaption of the intervention and recruitment of the participants to happen on schedule. Despite these concerns, the committee felt that the proposed rigorous study was likely to result in an intervention that could be of high impact.

DESCRIPTION (provided by applicant): Men who have sex with men (MSM) are disproportionally affected by HIV and substance abuse in the U.S., Europe, and Latin America. However, as in the general population, identifying and engaging MSM with problematic substance use (PSU) into treatment is a significant challenge, which results in the great majority of MSM with PSU never receiving treatment. The Substance Abuse and Mental Health Services Administration (SAMHSA) recommends Screening (S), Brief Intervention (BI), and Referral to Treatment (RT; SBIRT) as an early intervention for non-treatment-seeking individuals with risky alcohol and drug use, as well as the timely referral to more intensive substance abuse treatment for those with substance use disorders. The need for such an intervention is particularly acute in Argentina and Latin America, where there is little implementation of evidence-based interventions for MSM with PSU. The proposed study seeks to address this glaring gap by developing and piloting an electronic-SBI program tailored for MSM awaiting their HIV test at Nexo Asociacion Civil, our community partners in Buenos Aires. The tablet-based e-SBI will integrate substance use and sexual risk behavior screeners and individually tailored Motivational Interviewing (MI) as the BI. We will also adapt and pilot the implementation of the Young Men's Health Project (YMHP, now MHP since it will not be limited to young MSM), a four-session MI-based intervention that effectively reduced substance use and condomless anal intercourse among substance using MSM, as a brief treatment provided at Nexo for participants with moderate or high-risk substance use. The Specific Aims of this R34 study are to: 1) develop a tailored e-SBI intervention for MSM in an HIV testing context aimed at reducing substance use and HIV risk behavior (Primary); 2) assess the feasibility and acceptability of integrating e-SBI into the HIV testing process at Nexo as measured by a) percent of MSM testing clients at Nexo who accept entry into the study; b) percent of participants who complete e-SBI; c) e-SBI acceptability ratings (Primary); 3) assess the feasibility and acceptability of implementing adapted YMHP at Nexo, as measured by a) the percent of MSM with moderate or high-risk substance use who enter and/or complete MHP; b) acceptability of MHP among those who received it; and c) percentage of sessions conducted by each MHP counselor that meets criteria for MI fidelity (Secondary). In Stage 1 (Development) we will develop e-SBI, adapt YMHP into MHP, train MHP counselors, and pilot e-SBI with 50 MSM coming to Nexo for HIV testing. In Stage 2 (RCT Pilot), we will randomize 200 MSM coming to Nexo for HIV testing at a 3:1 ratio (eSBI: Screening Assessments Only-SA) to assess the feasibility and acceptability of e-SBI among MSM coming to Nexo for HIV testing and establish and pilot the RCT process for a future trial. As a secondary aim, we will assess uptake, acceptability, and feasibility of delivering MHP to participants with low or moderate risk

substance use and subsequent referrals to substance abuse treatment among participants with high-risk substance use or dependence. Lastly, we will explore preliminary findings on substance use and sexual risk reduction outcomes.

PUBLIC HEALTH RELEVANCE: This study begins to address a critical need for interventions that reduce both problem substance use (PSU) and HIV risk behavior among MSM in Latin America by adapting two evidence-based interventions, e-SBI and YMHP, for MSM in Argentina seeking HIV testing. Given that many MSM seek HIV testing in LGBT community agencies, these interventions have the potential to reach large numbers of MSM, help them reflect on their use of drugs and alcohol, and help link those with PSU to care. If proven efficacious in a future RCT, the implementation of these intervention in such agencies will help address a key contributor to HIV risk behavior and infection as well as reduce drug and alcohol use among MSM, who are disproportionately affected by HIV and substance use.

CRITIQUE 1

Significance: 3
Investigator(s): 2
Innovation: 4
Approach: 2
Environment: 1

Overall Impact: This proposal addresses a significant issue at the intersection of HIV prevention and substance misuse among MSM in Argentina. The overall impact would be high if the intervention is successful and widely implemented in Argentina. However, uptake in Argentina is very low and the proposal did not address this issue. The project is not innovative, other than adapting a well-established and researched intervention to a different setting. The research team is outstanding with robust research programs. The approach is strong including a robust and appropriate intervention development and adaptation plan. They will use a CAB to contextualize the intervention and will collect mixed data with surveys and focus groups. The adapted intervention will undergo a randomized controlled trial with 200 MSM. Participants will be followed for 6 months. The plan is complex but feasible given the teams' experience and prior work. The environment is outstanding with resources, established partnerships, and access to the population. In conclusion, despite the lack of innovation and concerns about the intervention's uptake in Argentina, the potential overall impact of this project remains high because it addresses a significant health problem and deploys a rigorous scientific plan.

1. Significance:

Strengths

- The study seeks to address a significant issue: Poly substance misuse and HIV risk behaviors among MSM in Argentina.
- Prior research supporting this application includes robust preliminary results that the efficacy of SBIRT and MI in reducing both substance misuse and HIV risk with other populations
- The intervention will be implemented in a CBO when MSM are waiting for the HIV test results. This may be an important place and time to increase their motivation to change their risk behaviors

Weaknesses

- While the studies will develop a tailored version of the eSBI intervention for MSM in Argentina, this new intervention won't substantially contribute to the SBIRT and MI literature that already exists.
- The applicant noted that the uptake of evidence-based interventions is difficult in Argentina. Even though this is a e-SBI, uptake still requires buy in and financial resources. There is no discussion about the sustainability of the intervention.

2. Investigator(s):

Strengths

- The research team has a demonstrated record of productive research in the field of HIV prevention, substance misuse, and LGBTQ+ populations
- PI has published multiple key papers on the proposed activity, including motivational interviewing, interventions manuals, substance misuse among MSM in Argentina
- Co-investigator Dr. Naar is a leader in the field of intervention science
- Mr. Marone, the local Project Director has prior experience working on a federally funded project in Argentina and significant experience with the study's population

Weaknesses

- The effort for Dr. Naar is low. That said, her experience and expertise is likely to enhance the quality of the study

3. Innovation:

Strengths

- Implementing the tailored eSBI and MHP interventions in HIV clinics and integrating them with HIV testing is innovative

Weaknesses

- Overall, this project is not very innovative. It tests evidence-based interventions that have already had a great deal of research support with myriad populations, including Latino MSM.

4. Approach:

Strengths

- The use of a CAB to assist with the development of measures and adaptation of the interventions will likely enhance their acceptability
- The research team has a solid plan to develop the e-SBI with the use of Computerized Intervention Authoring System, V.3 (CIAS 3.0) and User Centered Rapid Application Development process (UCRAD)
- The selected measures are standardized, well established and appropriate
- Mixed data will be collected and a data integration plan was included
- The analysis plan is rigorous and appropriate
- Follow up data will be collected over 6 months

- Alternative approached were discussed

Weaknesses

- None noted

5. Environment:

Strengths

- The environment at Florida State University and Nexo Asociacíon Civil in Argentina is outstanding because of established partnership, access to the study population, and access to scientific support

Weaknesses

- None noted

Study Timeline:

Strengths

- The timeline included all activities proposed
- The timeline seems feasible

Weaknesses

- None noted

Protections for Human Subjects:

Unacceptable Risks and/or Inadequate Protections

- The HS plan is thorough and provides a balanced analysis of risk and benefits
- Protections have been placed to minimize risks (e.g., training, use of de-identified data, password secured servers).
- One concern however: MI sessions will be video recorded and shown to the CAB. This creates issues about privacy and confidentiality as it is possible that CAB members may recognize a participant and hence hear information that the participant would rather keep private. There is no plan in place to address this risk.

Data and Safety Monitoring Plan (Applicable for Clinical Trials Only):

Acceptable

- o Includes staff training, use of HIPPA compliant software, IRB oversight, monitoring and reporting of adverse events

Inclusion Plans:

- Sex/Gender: Distribution not justified scientifically
- Race/Ethnicity: Distribution justified scientifically
- For NIH-Defined Phase III trials, Plans for valid design and analysis: Not applicable
- Inclusion/Exclusion Based on Age: Distribution justified scientifically

- There is no mention of the eligibility of people who do not identify either as male or female

Vertebrate Animals:
Not Applicable (No Vertebrate Animals)

Biohazards:
Not Applicable (No Biohazards)

Resource Sharing Plans:
Acceptable

- Will share de-identified data as appropriate

Budget and Period of Support:
Recommend as Requested

CRITIQUE 2

Significance: 2
Investigator(s): 2
Innovation: 3
Approach: 4
Environment: 1

Overall Impact: The study aims at developing and testing a relatively novel intervention to improve HIV prevention among men who have sex with men (MSM) with problematic substance use (PSU) in Buenos Aires, Argentina. The intervention to be tested is a tailored electronic Screening Brief Intervention (SBI) for MSM awaiting their HIV test. The study will be implemented with Nexos, a well-established community organization in Buenos Aires. As part of the aims of this R34-level study, the research team is aiming at assessing the feasibility and acceptability of integrating e-SBI into the HIV testing services at Nexo and assessing the feasibility and acceptability of implementing an adapted version of Young Men's Health Project (YMHP). The research team includes experts in the development of interventions – particularly interventions to increase HIV prevention and reduce the negative effects of PSU. Dr. Balan is bicultural and bilingual Latino scientist with extensive experience working with Latino MSM in the United States and Latin America. He has experience conducting similar research to the proposed in this study, including research in Argentina and in partnership with Nexos. The methodological expertise for the quantitative analysis will be guided by Dr. Leu, and this might be the weakest of all the collaborations proposed for this study. Overall, the implementation of this study could lead to the development of culturally congruent HIV and substance use prevention services. The design of the study is appropriate for the future development of a well-informed RCT.

1. Significance:

Strengths

- Latino MSM are disproportionally affected by the HIV epidemic and are also at increased risk for PSU. This trend is similar in Latin America and the data included in the proposal illustrate that MSM in Argentina could benefit from the intervention to be develop in this study.
- The interventions and strategies (i.e., SBIRT, YMHP, MI) to be integrated as part of the intervention package to be tested in this study have proven to be effective with similar populations. These interventions have also been adapted with some success among other populations, particularly in the continental United States.

Weaknesses

- There is limited evidence of the need of this approach to intervene with Latin American MSM. It is the use of the technologies proposed feasible and sustainable?
- There is very limited evidence of the use of YMHP with Latino or Spanish speaking MSM.

2. Investigator(s):

Strengths

- Dr. Balan has experience working with Latino MSM and MSM in Latin America, particularly Argentina. He also has experience working with Nexos and Mr. Marone.
- Dr. Naar has ample experience in the development of interventions and the use of MI for the prevention of HIV and PSU.

Weaknesses

- Besides Dr. Balan and Mr. Marone the team lacks expertise in the development and cultural adaptation of interventions with Latino populations, particularly Spanish-speaking MSM.

3. Innovation:

Strengths

- Implementation of evidence-based interventions for a population and in a context with a dearth of efficacious strategies for HIV and PSU prevention.
- Integration of screening for PSU in the context of HIV testing could provide to expand strategies or services for populations that are not traditionally served by HIV or primary care services.

Weaknesses

- The research team proposes integrating different interventions and strategies to address PSU and HIV risk behavior among MSM, but the acceptability of this kind of eHealth intervention is lacking.

4. Approach:

Strengths

- The RCT pilot is well designed and will provide very good information for the potential development of an RCT.
- The use of mixed methods in the development of the intervention should provide rich and detailed information.
- The development and adaptation of the interventions are guided by evidence-based approaches.

Weaknesses

- The timeline for the implementation is extremely ambitious (e.g., one moth for the cultural adaptation of the intervention MHP).
- There is no evidence of how the measures to be used during the "Pre-design and interface prototyping" (e.g., SIP-AD/MSM, SPAS) will be culturally (and linguistically) adapted or if these measures have been already tested with the study population or similar populations.
- While Nexos (community partner) provide HIV testing to many MSM over the year, there is no evidence that they can recruit participants who meet the inclusion criteria for the study (Using illicit drugs at least monthly and/or receiving a score >4 in the AUDIT C). Their ambitious goal for recruitment aimed at four participants per day, three days per week.

5. Environment:

Strengths

- The academic institution involved in the research project all well positioned to provide the resources and support for the implementation of the study.
- The research team is working with an organization abroad (Nexos) with previous experience collaborating with US-based scientist and in the implementation of NIH-funded studies.

Weaknesses

- None noted by reviewer.

Study Timeline:

Strengths

- Follow a logic approach of collaboration between the research team and the agency from where participants will be recruited.

Weaknesses

- The timeline is very ambitious. Only provides for 1 month for the development of the intervention, 1 month for the adaptation of YMP, and 2.5 month for recruitment of research participants.

Protections for Human Subjects:

Acceptable Risks and/or Adequate Protections

- A comprehensive plan for the protection of human subjects is included.

Data and Safety Monitoring Plan (Applicable for Clinical Trials Only):

Acceptable

- A reasonable plan is proposed for the RCT pilot.

Inclusion Plans:

- Sex/Gender: Distribution justified scientifically
- Race/Ethnicity: Distribution justified scientifically

- For NIH-Defined Phase III trials, Plans for valid design and analysis: Not applicable
- Inclusion/Exclusion Based on Age: Distribution justified scientifically
- The proposal includes clear scientific rationale for the human subjects to be included in the study.

Vertebrate Animals:
Not Applicable (No Vertebrate Animals)

Biohazards:
Not Applicable (No Biohazards)

Resource Sharing Plans:
Acceptable

- Research findings will be properly share with the scientific community.

Budget and Period of Support:
Recommend as Requested

CRITIQUE 3

Significance: 2
Investigator(s): 1
Innovation: 3
Approach: 3
Environment: 1

Overall Impact: Evidence based interventions that target substance use and HIV risk behavior among MSM are greatly needed in Latin America. The investigative team and research environments are excellent. The focus on using an evidence based intervention to reduce substance use and HIV risk behavior among MSM in Argentina is novel. Key aspects that contribute to rigor include: a well described and rigorous iterative development process for the app, a well described process for adapting the MI intervention, a rigorous counselor training and fidelity monitoring plan, and rigorous qualitative data coding and analysis. Rigor would be even further enhanced with the inclusion of an objective measure of drug use. The ambitious nature of the timeline does raise some feasibility concerns, particularly if there are unexpected delays in any of the study steps. Taken together, the overall impact level is deemed to be high.

1. Significance:
Strengths

- The premise for the proposed work is strong.
- Evidence based interventions to reduce HIV risk among MSM in Argentina and more broadly in Latin America are needed.

- Targeting both substance use and HIV risk behavior is logical.

Weaknesses

- The timeline may be overly ambitious for a 3-year study.

2. Investigator(s):

Strengths

- The investigative team is excellent.
- They have successfully completed similar work in the past, including at the proposed recruitment site in Argentina.

Weaknesses

- None noted by reviewer.

3. Innovation:

Strengths

- The most innovative aspect of the proposed work is the focus on using an evidence based intervention to reduce substance use and HIV risk behavior among MSM in Argentina.

Weaknesses

- Other aspects of the proposed intervention are not particularly novel.

4. Approach:

Strengths

- The level of methodological rigor is high.
- The investigators propose a well described and rigorous iterative development process for the app, incorporating input and feedback from stakeholders and users.
- The process for adapting the MI intervention is also well described.
- The proposed counselor training process is rigorous, as is the proposed plan to monitor fidelity.
- The methodology that will be used to code and analyze the qualitative data is also rigorous.
- The use of an authoring tool that does not require a programmer is a strength.
- Sex as a biological variable is appropriately considered given that the sole focus on MSM is well justified.

Weaknesses

- The incorporation of an objective measure of drug use would add to rigor.
- The ambitious nature of the proposed timeline does raise some feasibility concerns.

5. Environment:

Strengths

- The research environments are excellent and have supported similar work in the past.

Weaknesses

- None noted by reviewer.

Study Timeline:

Strengths

- The timeline is well delineated.

Weaknesses

- Given the large number of steps proposed for a 3-year study, the timeline may be overly ambitious particularly if there are unforeseen delays at any step.

Protections for Human Subjects:

Acceptable Risks and/or Adequate Protections

Data and Safety Monitoring Plan (Applicable for Clinical Trials Only):

Acceptable

Inclusion Plans:

- Sex/Gender: Distribution not justified scientifically
- Race/Ethnicity: Distribution justified scientifically
- For NIH-Defined Phase III trials, Plans for valid design and analysis: Not applicable
- Inclusion/Exclusion Based on Age: Distribution justified scientifically
- It is not clear whether they will limit their recruitment to cisgender men.

Vertebrate Animals:

Not Applicable (No Vertebrate Animals)

Biohazards:

Not Applicable (No Biohazards)

Resource Sharing Plans:

Acceptable

Budget and Period of Support:

Recommend as Requested

THE FOLLOWING SECTIONS WERE PREPARED BY THE SCIENTIFIC REVIEW OFFICER TO SUMMARIZE THE OUTCOME OF DISCUSSIONS OF THE REVIEW COMMITTEE, OR REVIEWERS' WRITTEN CRITIQUES, ON THE FOLLOWING ISSUES:

PROTECTION OF HUMAN SUBJECTS: ACCEPTABLE

INCLUSION OF WOMEN PLAN: ACCEPTABLE

INCLUSION OF MINORITIES PLAN: ACCEPTABLE

INCLUSION ACROSS THE LIFESPAN: ACCEPTABLE

COMMITTEE BUDGET RECOMMENDATIONS: The budget was recommended as requested.

Footnotes for 1 R34 DA055503-01; PI Name: Balan, Ivan C

NIH has modified its policy regarding the receipt of resubmissions (amended applications).See Guide Notice NOT-OD-18-197 at https://grants.nih.gov/grants/guide/notice-files/NOT-OD-18-197.html. The impact/priority score is calculated after discussion of an application by averaging the overall scores (1-9) given by all voting reviewers on the committee and multiplying by 10. The criterion scores are submitted prior to the meeting by the individual reviewers assigned to an application, and are not discussed specifically at the review meeting or calculated into the overall impact score. Some applications also receive a percentile ranking. For details on the review process, see http://grants.nih.gov/grants/peer_review_process.htm#scoring.

MEETING ROSTER

HIV/AIDS Intra- and Inter-personal Determinants and Behavioral Interventions Study Section
Risk, Prevention and Health Behavior Integrated Review Group
CENTER FOR SCIENTIFIC REVIEW
HIBI
07/12/2021 - 07/13/2021

Notice of NIH Policy to All Applicants: Meeting rosters are provided for information purposes only. Applicant investigators and institutional officials must not communicate directly with study section members about an application before or after the review. Failure to observe this policy will create a serious breach of integrity in the peer review process, and may lead to actions outlined in NOT-OD-14-073 at https://grants.nih.gov/grants/guide/notice-files/NOT-OD-14-073.html, NOT-OD-15-106 at https://grants.nih.gov/grants/guide/notice-files/NOT-OD-15-106.html, and NOT-OD-18-115 at https://grants.nih.gov/grants/guide/notice-files/NOT-OD-18-115.html, including removal of the application from immediate review.

CHAIRPERSON(S)

KIPKE, MICHELE D, PHD
PROFESSOR
DEPARTMENTS OF PEDIATRICS
AND PREVENTIVE MEDICINE
KECK SCHOOL OF MEDICINE
UNIVERSITY OF SOUTHERN CALIFORNIA
LOS ANGELES, CA 90028

MEMBERS

BUTLER, LISA MICHELLE, PHD
ASSOCIATE RESEARCH PROFESSOR
INSTITUTE FOR COLLABORATION ON HEALTH,
INTERVENTION, AND POLICY
UNIVERSITY OF CONNECTICUT
STORRS, CT 06269

COMULADA, WARREN SCOTT, DRPH
ASSOCIATE PROFESSOR
DEPARTMENT OF PSYCHIATRY
AND BIOBEHAVIORAL SCIENCES
SCHOOL OF PUBLIC HEALTH
UNIVERSITY OF CALIFORNIA, LOS ANGELES
LOS ANGELES, CA 90024

DODGE, BRIAN MARK, PHD
PROFESSOR
DEPARTMENT OF APPLIED HEALTH SCIENCE
INDIANA UNIVERSITY SCHOOL OF PUBLIC HEALTH
BLOOMINGTON, IN 47405

GRAHAM, SUSAN MARIE, MD, PHD
PROFESSOR
DIVISION OF ALLERGY AND INFECTIOUS DISEASES
DEPARTMENTS OF MEDICINE AND GLOBAL HEALTH
SCHOOL OF MEDICINE
UNIVERSITY OF WASHINGTON
SEATTLE, WA 98104

GROV, CHRISTIAN, PHD
PROFESSOR AND CHAIR
DEPARTMENT OF COMMUNITY HEALTH
AND SOCIAL SCIENCES
SCHOOL OF PUBLIC HEALTH AND HEALTH POLICY
CITY UNIVERSITY OF NEW YORK
NEW YORK, NY 10027

HANSEN, NATHAN B, PHD
DEPARTMENT HEAD AND PROFESSOR
DEPARTMENT OF HEALTH PROMOTION AND BEHAVIOR
COLLEGE OF PUBLIC HEALTH
UNIVERSITY OF GEORGIA
ATHENS, GA 30602

HORVATH, KEITH JOSEPH, PHD
ASSOCIATE PROFESSOR
DEPARTMENT OF CLINICAL PSYCHOLOGY
SAN DIEGO STATE UNIVERSITY
SAN DIEGO, CA 92120

IWELUNMOR, JULIET, PHD
ASSOCIATE PROFESSOR
DEPARTMENT OF BEHAVIORAL SCIENCE AND
HEALTH EDUCATION
COLLEGE FOR PUBLIC HEALTH AND SOCIAL JUSTICE
ST. LOUIS UNIVERSITY
ST. LOUIS, MO 63104

JOHNSON, DAWN M, PHD *
ASSOCIATE PROFESSOR
DEPARTMENT OF PSYCHOLOGY
UNIVERSITY OF AKRON
AKRON, OH 44325

LIPPMAN, SHERI ANN, PHD *
ASSOCIATE PROFESSOR
CENTER FOR AIDS PREVENTION STUDIES
UNIVERSITY OF CALIFORNIA, SAN FRANCISCO
SAN FRANCISCO, CA 94143

LOVEJOY, TRAVIS IAN, PHD
ASSOCIATE PROFESSOR
DEPARTMENT OF PSYCHIATRY
SCHOOL OF MEDICINE
OREGON HEALTH AND SCIENCE UNIVERSITY
PORTLAND, OR 97239

MACDONELL, KAREN KOLMODIN, PHD *
ASSOCIATE PROFESSOR
DEPARTMENT OF FAMILY MEDICINE
AND PUBLIC HEALTH SCIENCES
SCHOOL OF MEDICINE
WAYNE STATE UNIVERSITY
DETROIT, MI 48202

MUESSIG, KATHRYN E, PHD *
ASSISTANT PROFESSOR
DEPARTMENT OF HEALTH BEHAVIOR
GILLINGS SCHOOL OF GLOBAL PUBLIC HEALTH
UNIVERSITY OF NORTH CAROLINA AT CHAPEL HILL
CHAPEL HILL, NC 27599

OWCZARZAK, JILL, PHD *
ASSOCIATE PROFESSOR
DEPARTMENT OF HEALTH, BEHAVIOR AND SOCIETY
BLOOMBERG SCHOOL OF PUBLIC HEALTH
JOHNS HOPKINS UNIVERSITY
BALTIMORE, MD 21205

PATEL, VIRAJ V, MD, MPH *
ASSOCIATE PROFESSOR
DEPARTMENT OF MEDICINE
ALBERT EINSTEIN COLLEGE OF MEDICINE
BRONX, NY 10461

RAEL, CHRISTINE TAGLIAFERRI, PHD *
ASSISTANT PROFESSOR
COLLEGE OF NURSING
UNIVERSITY OF COLORADO ANSCHUTZ MEDICAL CAMPUS
AURORA, CO 80045

RAMSEY, SUSAN E, PHD
ASSOCIATE PROFESSOR
DIVISION OF GENERAL INTERNAL MEDICINE
RHODE ISLAND HOSPITAL
BROWN UNIVERSITY
PROVIDENCE, RI 02903

RODRIGUEZ-DIAZ, CARLOS EMANUEL, PHD *
ASSOCIATE PROFESSOR
DEPARTMENT OF PREVENTION AND COMMUNITY HEALTH
MILKEN INSTITUTE SCHOOL OF PUBLIC HEALTH
THE GEORGE WASHINGTON UNIVERSITY
WASHINGTON, DC 20052

SAFREN, STEVEN A, PHD
PROFESSOR
DEPARTMENT OF PSYCHOLOGY
COLLEGE OF ARTS AND SCIENCES
UNIVERSITY OF MIAMI
CORAL GABLES, FL 33124

SICONOLFI, DANIEL, MPH, PHD *
BEHAVIORAL SCIENTIST
RAND CORPORATION
PITTSBURGH, PA 15213

SSEWAMALA, FRED M, PHD
PROFESSOR
INSTITUTE FOR PUBLIC HEALTH
BROWN SCHOOL
WASHINGTON UNIVERSITY
ST. LOUIS, MO 63130

STOCKMAN, JAMILA KINSHASA, PHD
PROFESSOR
DIVISION OF GLOBAL PUBLIC HEALTH
DEPARTMENT OF MEDICINE
SCHOOL OF MEDICINE
UNIVERSITY OF CALIFORNIA, SAN DIEGO
LA JOLLA, CA 92093

SULLIVAN, PATRICK SEAN, PHD
PROFESSOR
DEPARTMENT OF EPIDEMIOLOGY
ROLLINS SCHOOL OF PUBLIC HEALTH
EMORY UNIVERSITY
ATLANTA, GA 30322

TANNER, AMANDA E, MPH, PHD *
ASSOCIATE PROFESSOR
DEPARTMENT OF PUBLIC HEALTH EDUCATION
SCHOOL OF HEALTH AND HUMAN SCIENCES
UNIVERSITY OF NORTH CAROLINA GREENSBORO
GREENSBORO, NC 27402

THAMES, APRIL D, PHD
ASSOCIATE PROFESSOR
DEPARTMENT OF PSYCHOLOGY
UNIVERSITY OF SOUTHERN CALIFORNIA
LOS ANGELES, CA 90089

THIELMAN, NATHAN M, MD, MPH *
PROFESSOR
DEPARTMENT OF MEDICINE
DUKE UNIVERSITY SCHOOL OF MEDICINE
DURHAM, NC 27710

WEBEL, ALLISON R, PHD
PROFESSOR
SCHOOL OF NURSING
UNIVERSITY OF WASHINGTON
SEATTLE, WA 98195

WILSON, TRACEY ELIZABETH, PHD *
PROFESSOR
DEPARTMENT OF COMMUNITY HEALTH SCIENCES
SCHOOL OF PUBLIC HEALTH
DOWNSTATE MEDICAL CENTER
THE STATE UNIVERSITY OF NEW YORK
BROOKLYN, NY 11203

WILTON, LEO, PHD, MPH
PROFESSOR
DEPARTMENT OF HUMAN DEVELOPMENT
COLLEGE OF COMMUNITY AND PUBLIC AFFAIRS
BINGHAMTON UNIVERSITY
BINGHAMTON, NY 13902

WINDSOR, LILIANE CAMBRAIA, PHD
ASSOCIATE PROFESSOR
SCHOOL OF SOCIAL WORK
THE UNIVERSITY OF ILLINOIS AT URBANA-CHAMPAIGN
URBANA, IL 61801

YBARRA, MICHELE L., PHD, MPH *
CEO AND RESEARCH DIRECTOR
CENTER FOR INNOVATIVE PUBLIC HEALTH RESEARCH
SAN CLEMENTE, CA 92672

SCIENTIFIC REVIEW OFFICER

RUBERT, MARK P, PHD
SCIENTIFIC REVIEW OFFICER
CENTER FOR SCIENTIFIC REVIEW
NATIONAL INSTITUTES OF HEALTH
BETHESDA, MD 20892

EXTRAMURAL SUPPORT ASSISTANT

CAMBRELEN, AMY ANGELA
EXTRAMURAL SUPPORT ASSISTANT
CENTER FOR SCIENTIFIC REVIEW
NATIONAL INSTITUTE OF HEALTH
BETHESDA, MD 20892

* Temporary Member. For grant applications, temporary members may participate in the entire meeting or may review only selected applications as needed.

Consultants are required to absent themselves from the room during the review of any application if their presence would constitute or appear to constitute a conflict of interest.
